# Training Experience and Family Interactions in Care Settings: Findings from the National Dementia Workforce Study

**DOI:** 10.1093/geroni/igaf122.3675

**Published:** 2025-12-31

**Authors:** Sunshine Rote, Heehyul Moon, Kathryn Williams-Sites

**Affiliations:** University of Louisville, Louisville, Kentucky, United States; University of Louisville, Louisville, Kentucky, United States; University of Louisville, Louisville, Kentucky, United States

## Abstract

This study analyzes recently released data from the National Dementia Workforce Study (NDWS). The NDSW is the first large, annual survey of the dementia care workforce in the United States and sponsored by the NIA/NIH. Our analysis examines the relationships between training experiences, family interactions, and worker confidence among direct care workers in nursing home (n = 390) and assisted living facility settings (n = 445). Over 90% of direct care workers in both settings report formal or informal training in dementia, injury prevention, and responding to the needs of residents, while the largest informal and formal training gaps identified were in content related to working with families of people living with dementia. Bivariate analyses show that direct care workers with extensive formal and informal, on-the-job training report significantly greater confidence in the ability to adapt to the changing needs of residents living with dementia compared to those with less training in both nursing homes and assisted living facilities. Direct care workers who report problems with residents’ family members are significantly less confident in their ability to remain motivated in providing care. These findings suggest that comprehensive training initiatives combined with family relationship management strategies may be essential for optimizing dementia care quality and worker retention in long-term care settings.

